# Is the measurement of the size of uterine lesions with positron emission tomography consistent in pre- and postmenopausal periods in endometrioid-type endometrial cancer?

**DOI:** 10.4274/tjod.64188

**Published:** 2018-03-29

**Authors:** Varol Gülseren, Mustafa Kocaer, Özgü Çelikkol Güngördük, İsa Aykut Özdemir, Muzaffer Sancı, Kemal Güngördük

**Affiliations:** 1 Kaman State Hospital, Clinic of Obstetrics and Gynecology, Kırşehir, Turkey; 2 University of Health Sciences, Clinic of Gynecologic Oncology, İzmir, Turkey; 3 Muğla Sıtkı Koçman University, Training and Research Hospital, Department of Gynecology and Oncology, Muğla, Turkey; 4 Bakırköy Dr. Sadi Konuk Training and Research Hospital, Clinic of Gynecology and Oncology, İstanbul, Turkey

**Keywords:** Positron emission tomography/computed tomography, endometrial cancer, premenopausal and reproductive periods

## Abstract

**Objective:**

We aimed to investigate the correlation of the size and volume of uterine tumors obtained using positron emission tomography/computed tomography (PET/CT) and pathology specimens in patients with endometrioid-type endometrial cancer (EEC) in the premenopausal period, and to compare the results with those of postmenopausal women. In the premenopausal period, the endometrium uses more glucose than in the postmenopausal period. Therefore, the measurement of uterine tumor size using PET/CT in the premenopausal period may normally be different.

**Materials and Methods:**

In this retrospective study, we reviewed the records of patients who were diagnosed as having EEC and underwent hysterectomy. Only patients who underwent preoperative PET/CT imaging were included in the study. The thickness and volume of the uterine lesion, and its maximum standardized uptake value as obtained using PET/CT and hysterectomy pathology specimens were recorded.

**Results:**

Tumor size (p=0.051) and volume (p=0.404) were not found to be correlated with the imaging method used in premenopausal women and pathologic specimens. However, there was a correlation in postmenopausal women (p<0.001 for tumor size and p<0.001 for tumor volume). PET/CT has higher sensitivity, specificity, and positive predictive value in the postmenopausal period in the detection of >20 mm uterine tumors.

**Conclusion:**

PET/CT has a limited role in the measurement of the size of uterine lesions in all patients, especially in the premenopausal period; therefore, we recommend that frozen-section examinations be used intraoperatively to decide on lymph node dissection.

**PRECIS:** Positron emission tomography/computed tomography does not accurately assess the size of uterine lesions due to physiologic events in the endometrium and uterus in reproductive ages.

## Introduction

Endometrial cancer is the most common gynecologic malignancy in developed countries^(1,2,3)^. Prognosis is affected by the age of the patient, histologic type and grade of the tumor, cervical invasion, depth of myometrial invasion, lymph node involvement, and distant organ metastasis^(1,2)^. Fluorine-18 (^18^F) fluorodeoxyglucose (FDG) positron emission tomography/computed tomography (PET/CT) is an imaging modality used to obtain anatomic and metabolic data on cancer cells in numerous malignancies^(2,3)^. It is helpful to evaluate tumor perfusion and metabolism screening using the following radioisotopes: carbon-11, ^18^F, nitrogen-13, oxygen-15 and rubidium-82^(4)^. Of these, ^18^F-FDG passes through the cell membrane in the same way as glucose and is effectively trapped when it is phosphorylated and cannot be metabolized by the following enzyme: phosphofructokinase-1. Thus, ^18^F-FDG remaining within the cell reflects glucose uptake into the cell^(4)^. The standardized uptake value (SUV) is accepted as an indicator of tumor aggressiveness and a marker for metabolic alterations in cancer tissues^(2,3,4)^. The maximum SUV (SUV_max_) has been associated with the tumor proliferation rate, tumor grade, and expression of glucose transporters^(2,3,4)^. About 25% of patients with endometrioid-type endometrial cancer (EEC) are in the premenopausal periods^(5)^. In women of the premenopausal period, physiologic FDG accumulation in the uterus should be considered when focal FDG accumulation is observed in the pelvis^(6)^. In the endometrium, normal uptake of ^18^F-FDG PET/CT in patients who are premenopausal varies cyclically and increases in the ovulatory and menstrual phases^(7)^. In the premenopausal period, the endometrium consumes constant energy for proliferation and the secretory phases^(6)^. In the present study, we aimed to investigate the correlation of the size and volume of uterine tumors obtained using PET/CT and pathology specimens in patients with EEC in the premenopausal period and to compare the results with those of postmenopausal women.

## Materials and Methods

**Figure 1:**
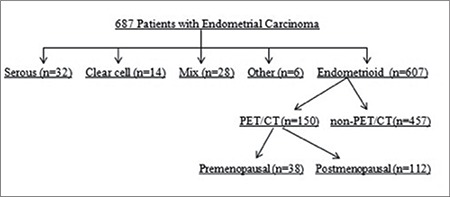
Flowchart of the study *PET/CT: Positron emission tomography/computed tomography*

In this retrospective study, we reviewed the records of patients who were diagnosed as having EEC and underwent hysterectomy at the Tepecik Training and Research Hospital, Clinic of Gynecologic Oncology between January 2012 and August 2016. Only patients who underwent preoperative ^18^F-FDG PET/CT imaging were included in the study. A flowchart of the study is shown in Figure 1. Diagnosis was confirmed histopathologically in all patients. The thickness and volume of the uterine lesion and its SUV_max_ value as obtained using ^18^F-FDG PET/CT and hysterectomy pathology specimens were recorded. Data including age, menopausal status, and comorbidities were recorded. Tumor staging was performed based on the International Federation of Gynecology and Obstetrics (FIGO) 2009 staging criteria^(8)^. The study was approved by the local ethics committee (Katip Çelebi University, approval number: 45, Date: 27/02/2014). Written informed consent was obtained from each patient. The study was conducted in accordance with the principles of the Declaration of Helsinki. All surgical specimens were evaluated by specialized gynecologic pathologists. The inclusion criteria were as follows: 1) all types of histology, 2) no intraoperative evidence of extrauterine spread, 3) performance of pelvic and para-aortic lymphadenectomy, and 4) histopathologically proven cervical stromal involvement. Uterine sections were selected from anterior and posterior aspects of the cervix, lower uterine segment, and uterine corpus. A minimum of 6 sections including a section of the deepest tumoral invasion was obtained for all specimens. Whole-body ^18^F-FDG PET/CT images were performed using a PET/CT scanner (Philips Gemini TF; Philips Healthcare, Andover, MA, USA), which consisted of a dedicated lutetium orthosilicate full-ring PET scanner and 16-slice CT. Both PET and low-dose CT scanning covered the skull to the proximal thigh. The protocol included 6 h of fasting before image acquisition, and all patients were asked to void before undergoing scanning. On the day of the examination, the serum glucose levels measured before ^18^F-FDG injections were found to be less than 140 mg/dL. Subsequently, ^18^F-FDG (6.5-13.4 µCi) was given intravenously 60 to 120 min before the CT scan, and the patients were instructed to rest in a semi-dark, temperate room between the injection and scanning. At 60 min after the administration of ^18^F-FDG, low-dose CT (50 mAs, 120 kV) covering the area from scull to the proximal thighs was performed to attenuate the correction and precise anatomic localization. An emission scan was then conducted in the three-dimensional mode. All images were reconstructed and stored as axial, coronal, and sagittal slices. The total scanning time was about 20 min per patient. The SUV_max_ was estimated for each hypermetabolic lesion.

### Statistical Analysis

This study was calculated to have 94% power and 71% effect size using the G power analysis program (Faul, Erdfelder, Lang and Buchner, 2007; version 3.0). Statistical analysis was performed using the Med-Calc for Windows version 16.0 statistical software (MedCalc Software, Mariakerke, Belgium). Descriptive data are expressed in mean ± standard deviation and percentages. Student’s t-test was used to compare the mean values between two independent groups, and the chi-square (χ^2^) test was used to compare nominal values between the two groups. Correlation analysis was performed using bivariate correlation analysis. The sensitivity, specificity, negative and positive predictive values of the ^18^F-FDG PET/CT were also calculated. Receiver operating characteristic (ROC) curve analysis was used to determine the optimal cut-off value of predictive tumor size >2 cm in uterin lesion with EEC. A p value of <0.05 was considered statistically significant.

## Results

Of all patients with EEC who underwent ^18^F-FDG PET/CT, 38 women were premenopausal, and 112 were postmenopausal. The demographic and clinical characteristics of the patients are shown in Table 1.

**Figure 2:**
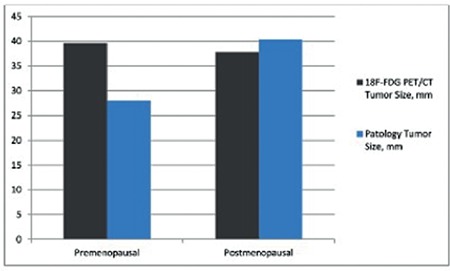
Correlation analysis* between ^18^F-FDG PET/CT and pathology report of tumor size ^18^F-FDG PET/CT: 18-Florin-Fluorodeoxyglucose-positron emission tomography/computed tomography, *: Spearman’s correlation analysis, p=0.051 for premenopausal, p<0.001 for postmenopausal

**Figure 3:**
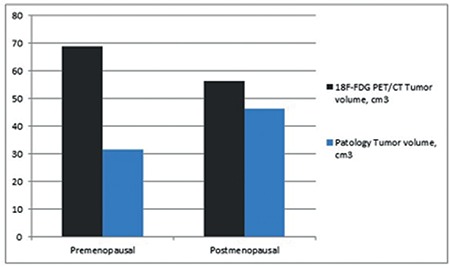
Correlation analysis* between ^18^F-FDG PET/CT and pathology report of tumor volume ^18^F-FDG PET/CT: 18 Florin-Fluorodeoxyglucose positron emission tomography/computed tomography, *: Spearman’s correlation analysis, p=0.404 for premenopausal, p<0.001 for postmenopausal

**Figure 4:**
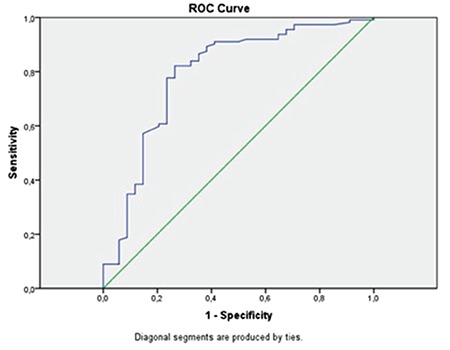
ROC curve associated with the maximum standardized uptake value to identify patients with tumor size >20 mm. The area under the curve was 0.791 (p<0.001) *SUV_max_: The maximum standardized uptake value*

The largest tumor size and total volume of both premenopausal and postmenopausal patients in the ^18^F-FDG PET/CT reports were compared with the pathology reports. The correlation analysis results are shown in Figures 2 and 3. The tumor size and volume were not found to be correlated with the imaging method used in premenopausal women and pathologic specimens for tumor size and tumor volume (p=0.051, correlation coefficient: 0.319; p=0.404, correlation coefficient: 0.139, respectively). However, there was a correlation in postmenopausal women for tumor size and tumor volume (p<0.001, correlation coefficient: 0.772; p<0.001 and correlation coefficient: 0.695, respectively). Sensitivity and specificity tests were performed in the premenopausal women and postmenopausal women by dividing the tumor size into the two groups (≤20 mm; >20 mm) in both ^18^F-FDG PET/CT reports and pathology specimens. In the former group, the sensitivity of ^18^F-FDG PET/CT to detect >20 mm tumors was 19/21 (90.4%), specificity was 6/17 (35.2%), the negative predictive value was 6/8 (75.0%), and the positive predictive value was 19/30 (63.3%). The pooled diagnostic indices to detect tumors >20 mm in postmenopausal women were as follows: sensitivity 88/94 (93.6%), specificity 11/18 (61.1%), negative predictive value 11/17 (64.7%), and positive predictive value 88/95 (92.6%). The optimal SUV_max_ value was investigated using ROC analysis to distinguish patients with tumor size >2 cm. The ROC analysis is shown in Figure 4 (p<0.001, area under the curve=0.791). SUV_max_ values of 10.5 and above were found as 82.1% sensitivity and 73.5% specificity for tumors with tumor size >2 cm. In addition, the ^18^F-FDG PET/CT showed 4/5 (80.0%) sensitivity, 28/33 (84.8%) specificity, 28/29 (96.6%) negative predictive value, and 4/9 (44.4%) positive predictive value to detect lymph node involvement in premenopausal women. In the postmenopausal period, however, the pooled diagnostic indices for lymph node involvement were as follows: sensitivity 12/15 (80.0%), specificity 91/97 (93.8%), negative predictive value 94/97 (96.9%), and positive predictive value 9/15 (60.0%).

## Discussion

In this retrospective study, we evaluated the accuracy of ^18^F-FDG PET/CT in the assessment of the size and volume of uterine lesions associated with EEC in premenopausal and postmenopausal patients with EEC. Based on our study results, we found that tumor volume and size were correlated in postmenopausal women, but not in premenopausal women. Previous studies showed that PET/CT had 81.8% sensitivity and 89.8% specificity in the detection of primary uterine tumors in patients with EEC^(9)^. In our study, the sensitivity of ^18^F-FDG PET/CT for detecting tumors >20 mm in premenopausal women was 90.4%, specificity was 35.2%, and the negative and positive predictive values were 75.0% and 63.3%, respectively. In the postmenopausal period, the sensitivity of ^18^F-FDG PET/CT to detect >20 mm tumors was 93.6%, specificity was 61.1%, and the negative and positive predictive values were 64.7% and 92.6%, respectively. The proposed cut-offs for SUV_max_ for these parameters to identify deep myometrial invasion in the literature is a relatively wide range, 9-18^(8,10)^. There was a significant association reported between the SUV_max_ of the primary tumors and maximum tumor size (p=0.001), but not between the SUV_max_ and menopause state (p=0.522)^(11)^. In our cohort, SUV_max_ >10.5 had 82.1% sensitivity and 73.5% specificity for tumors >2 cm. The ^18^F-FDG PET/CT imaging modality uses the intracellular glucose metabolism of tumor cells^(4)^. In the premenopausal period, the endometrium uses different amounts of glucose for menstruation, proliferation, ovulation, and secretion processes; however, the endometrium in the postmenopausal period uses less glucose^(6)^. In a study of endometrial ^18^F-FDG uptake in gynecologic malignancies in premenopausal women by Lerman et al.^(7)^ the mean SUV values were 5.0±3.2 in the menstrual phase, 2.6±1.1 in the proliferation phase, 3.7±0.9 in the ovulation phase, and 2.5±1.1 in the secretory phase (p<0.001). In the aforementioned study, the mean SUV value of the patients with abnormal cycles was 3.4±1.4 in patients with oligomenorrhea and 1.9±1.2 in patients with amenorrhea (p=0.02). PET may be influenced by tissue type^(9)^. The efficacy of PET/CT may be affected by the size of the tumor, and thus PET/CT may be limited for the detection of small tumors^(9)^. Furthermore, oral contraceptive use has been shown to affect the ^18^F-FDG uptake in the endometrium^(12)^. In our study, we hypothesized that ^18^F-FDG uptake in premenopausal women and the calculated tumor size and volume would be less correlated with the pathology specimens compared with postmenopausal women. In our study population, the calculated tumor size (p=0.051) and volume (p=0.404) on ^18^F-FDG PET/CT imaging in premenopausal women were not correlated with the pathology specimens. However, in the postmenopausal period, tumor size (p<0.001) and volume (p<0.001) on the ^18^F-FDG PET/CT scan were found to correlate with the pathology specimens. In addition, the sensitivity and specificity of the PET/CT to detect lymph node metastasis in premenopausal women was 80.0% and 84.8%, respectively, compared with 80.0% and 93.8% in postmenopausal women, respectively. The sensitivity was 40.9% in micrometastatic lymph nodes with (metastasis >2 mm) and 52.9% in those with (metastasis >5 mm)^(9)^. The sensitivity and specificity of PET/CT for detecting nodal metastases were 51.1-78.6% and 98.4-99.8%, respectively^(13,14)^. Although the sensitivity and specificity values for detecting lymph node involvement are similar in pre- and postmenopausal women, estimating the size of primary uterine lesions showed limited correlation in premenopausal women. In our cohort, there was no statistically significant difference between the SUV_max_ values of pre- and postmenopausal period uterine lesions. Therefore, we consider that PET/CT has a limited role in deciding for lymph node dissection in premenopausal women, and frozen-section examinations should be performed during surgery. In our cohort, we found that there were statistically significantly more patients with diabetes among the postmenopausal patients (p=0.005). However, previous studies reported that PET/CT could be applied to diabetics^(15)^. In all patients (diabetics and non-diabetics), the serum glucose levels measured before ^18^F-FDG injections were found to be less than 140 mg/dL.

### Study Limitations

Nonetheless, there are some limitations to this study. First, the study has a retrospective design. Second, the sample size is relatively small. Third, the endometrial phases of premenopausal women are still missing aspects of the study. Despite these limitations, the similarities of the demographic characteristics in the study population and analysis reports of the expert pathologists and radiologists increased the validity of our results and diminished the weaknesses. However, further large-scale, prospective studies are required to shed light on the role of ^18^F-FDG PET/CT in EEC.

## Conclusion

In conclusion, our study results suggest that ^18^F-FDG PET/CT has a limited role in the measurement of the size of the uterine lesion in all patients, especially in the premenopausal period; therefore, we recommend that frozen-section examinations should be performed intraoperatively to decide on lymph node dissection.

## Figures and Tables

**Table 1:**
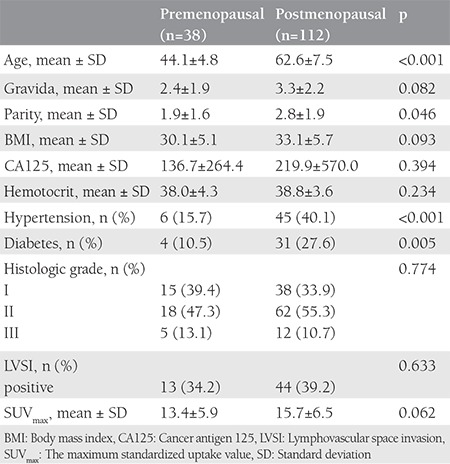
Demographic characteristics and clinical characteristics of the patients
